# Vaccines to Prevent Coccidioidomycosis: A Gene-Deletion Mutant of Coccidioides Posadasii as a Viable Candidate for Human Trials

**DOI:** 10.3390/jof8080838

**Published:** 2022-08-10

**Authors:** John N. Galgiani, Lisa F. Shubitz, Marc J. Orbach, M. Alejandra Mandel, Daniel A. Powell, Bruce S. Klein, Edward J. Robb, Mana Ohkura, Devin J. Seka, Thomas M. Tomasiak, Thomas P. Monath

**Affiliations:** 1 Valley Fever Center for Excellence, University of Arizona College of Medicine-Tucson, Tucson, AZ 85724, USA; 2 Department of Medicine, University of Arizona College of Medicine-Tucson, Tucson, AZ 85724, USA; 3 Department of Immunobiology, University of Arizona College of Medicine-Tucson, Tucson, AZ 85724, USA; 4 Bio5 Institute, University of Arizona, Tucson, AZ 85721, USA; 5 School of Plant Sciences, College of Agriculture and Life Sciences, University of Arizona, Tucson, AZ 85721, USA; 6 Department of Pediatrics, University of Wisconsin School of Medicine and Public Health, University of Wisconsin-Madison, Madison, WI 53706, USA; 7 Department of Internal Medicine, University of Wisconsin School of Medicine and Public Health, University of Wisconsin-Madison, Madison, WI 53706, USA; 8 Department of Medical Microbiology and Immunology, University of Wisconsin School of Medicine and Public Health, University of Wisconsin-Madison, Madison, WI 53706, USA; 9 Anivive Lifesciences, Long Beach, CA 90807, USA; 10 Department of Botany and Plant Pathology, College of Agricultural Sciences, Oregon State University, Corvallis, OR 97331, USA; 11 Department of Chemistry and Biochemistry, College of Science, University of Arizona, Tucson, AZ 85721, USA; 12 Crozet Biopharma LLC, Lexington, MA 02420, USA

**Keywords:** coccidioidomycosis, live-attenuate vaccines, cellular immunity, canines

## Abstract

Coccidioidomycosis is an endemic fungal infection that is reported in up to 20,000 persons per year and has an economic impact close to $1.5 billion. Natural infection virtually always confers protection from future exposure, and this suggests that a preventative vaccine strategy is likely to succeed. We here review progress toward that objective. There has been ongoing research to discover a coccidioidal vaccine over the past seven decades, including one phase III clinical trial, but for reasons of either efficacy or feasibility, a safe and effective vaccine has not yet been developed. This review first summarizes the past research to develop a coccidioidal vaccine. It then details the evidence that supports a live, gene-deletion vaccine candidate as suitable for further development as both a veterinary and a human clinical product. Finally, a plausible vaccine development plan is described which would be applicable to this vaccine candidate and also useful to other future candidates. The public health and economic impact of coccidioidomycosis fully justifies a public private partnership for vaccine development, and the development of a vaccine for this orphan disease will likely require some degree of public funding.

## 1. Introduction

Of the estimated 5.1 million fungal species [1], only a very few are pathogenic for mammals. Of those that cause disease in humans, most are opportunistic pathogens, exploiting breaches in host defenses of the skin or the immune system. Dimorphic endemic fungi such as *Blastomyces dermatiditis*, *Coccidioides* species, and *Histoplasma capsulatum*, while more virulent in immunocompromised patients [2,3,4,5,6], also produce illness in humans that appear to be otherwise immunologically normal.

An excellent review was recently published detailing the past efforts to prevent fungal infections generally through vaccination and the challenges that still lie ahead [7]. Since then, too recent to be included in that review, considerable progress has been made in vaccine development and human clinical trials for one specific fungal disease, coccidioidomycosis (CM). These advances have been based upon the discovery of a gene-deletion mutant of *C. posadasii* whose virulence is extremely attenuated and which, as a vaccine, produces very broad protection against subsequent coccidioidal infection. This review will summarize the decades-long search for a coccidioidal vaccine and then the new developments which provide a firm rationale for resuming human clinical trials.

## 2. The Case for Vaccines to Prevent Coccidioidomycosis

The excellent recent general review of fungal vaccines [7] includes CM as one of the prime diseases that might benefit from preventive immunization. CM, endemic to many parts of the Western Hemisphere [8], especially Arizona and Southern California [9], is clearly important regionally and is occasionally exported elsewhere because of tourism and business travel [10,11]. In the United States, since 2010, CM reported to the Centers for Disease Control (CDC) has ranged from 10,000 to over 20,000 cases annually. However, clinically significant illness is frequently undiagnosed because specific laboratory testing is often not performed [12,13] or because tests performed early in the illness are falsely negative [14], resulting in prescribing of ineffective antibiotics. A recent report from a large urgent care group in Phoenix and Tucson, Arizona showed that in 2018 and 2019 less than 7% of patients with pneumonia were tested for CM. In the past two years and with a targeted education campaign, this rate has improved, but more than three-quarters of the pneumonia patients have still not been tested (Pu et al. presented at the Coccidioidomycosis Study Group, 2022, Bakersfield, CA, USA). These findings are in keeping with preliminary CDC estimates that the extent of underreporting ranges from six to 14 times [15].

The most common coccidioidal illness is a community acquired pneumonia, frequently occurring with musculoskeletal pain, rashes, and protracted fatigue. Although this syndrome is usually self-limited, it causes significant morbidity lasting from many weeks to many months [16,17]. A small percentage of CM infections produce progressive tissue destruction, either as a chronic fibrocavitary pneumonia or with hematogenously disseminated infection beyond the chest to involve the central nervous system, skin, or skeleton. These complications disproportionately impact the immunosuppressed, diabetics, persons of color, pregnant women, and the elderly [18]. Antifungal therapies are available that suppress active coccidioidal infections, but none are curative. Some patients, such as those with coccidioidal meningitis, must receive lifelong treatment. The annual economic impact of CM has been estimated to range from $385 million in direct costs nationally [19] to nearly $1.5 billion in total lifetime costs in Arizona and California [20,21].

**Figure 1:**
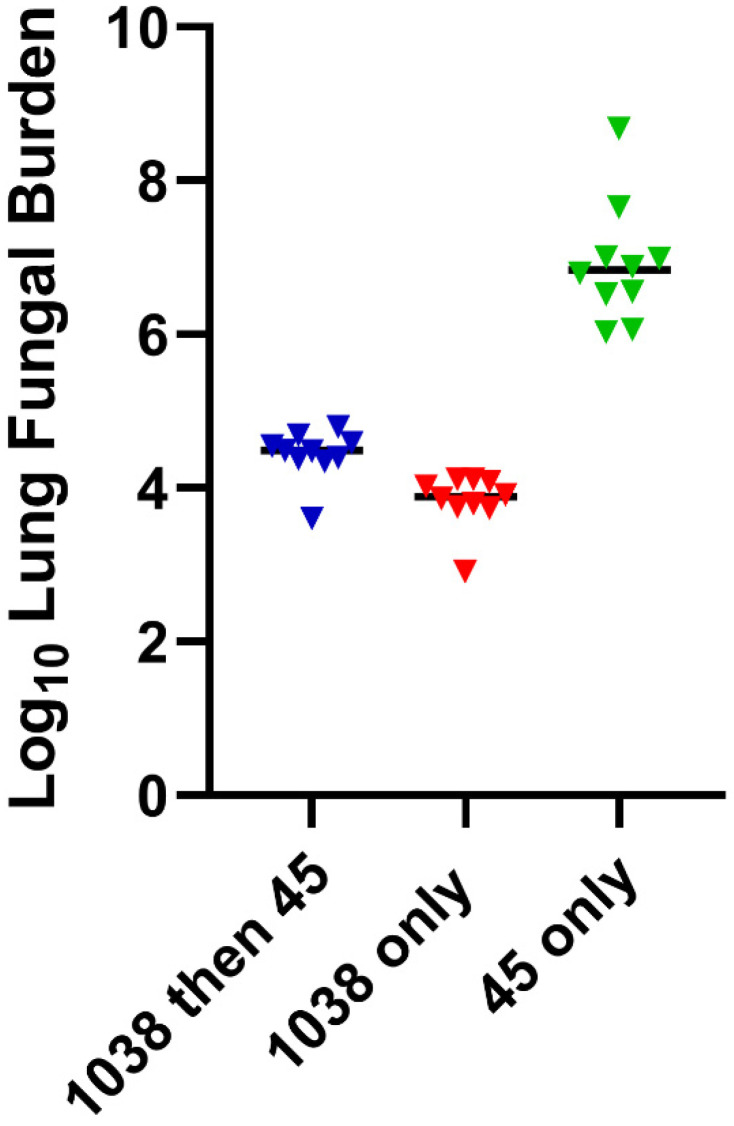
C57BL/6 × DBA/2 F1 mice were infected with *C. posadasii* strain 1038, a lower virulence clinical isolate, and Strain 45, which contains an ectopic insertion of the hygromycin resistance gene *hphb* and is lethal to mice. The hygromycin allows separate detection of 1038 (hygromycin sensitive) and Strain 45 (hygromycin resistant). Two groups of mice were infected IN with 1038 and one group was given saline. Six weeks later, the saline and one 1038-infected group were given strain 45 IN at approximately 3× the lethal dose. In mice given strain 45 following 1038, no growth of the second strain was detected on agar plates containing hygromycin, and lung fungal burdens were similar for strain 1038 in both groups, while mice given 45 only were dead by 16 days with high lung fungal burdens. Thus, initial infection with a low virulence strain protects from a second highly virulent infection.

All evidence to date indicates that infection following the inhalation of a coccidioidal spore (arthroconidium) produces life-long resistance to a second illness from inhaling another arthroconidium [22]. This is virtually always the case for persons who were not clinically diagnosed with CM and identified only by dermal hypersensitivity to a coccidioidal skin test [23] or for those with self-resolved uncomplicated infections [24]. Moreover, experimental infection with coccidioidal strains of low virulence for mice have been shown to produce resistance to subsequent coccidioidal infection with either *C. posadasii*, the Silveira strain, or with *C. immitis*, strain RS, both highly virulent strains for mice [25,26] (Figure 1). These observations form the rationale that vaccination might also be able to be protective, particularly with a live-attenuated vaccine that mimics natural immunity.

## 3. Past Attempts to Create a Coccidioidal Vaccine

Efforts to develop a preventative coccidioidal vaccine have been underway since the 1950s [27]. Discovery of media that supported growth of spherules in vitro [28,29,30] and adapting strain Silveira to propagate as spherules in continuous culture [31] resulted in studies demonstrating protection in mice against intranasal coccidioidal infection by vaccination with formalin-killed spherules (FKS) [25,32,33]. Numerous pre-clinical studies [34] led to Phase I clinical studies [35,36] and eventually a Phase III randomized field trial which demonstrated that FKS vaccination resulted in little if any protection against illness produced by subsequent natural coccidioidal infection [37]. Moreover, FKS produced very significant injection site reactions which further discouraged the continued pursuit of this material as a vaccine candidate.

That a search for a protective vaccine against CM continued, despite this setback, reflects just how strong the interest was, particularly in the California’s Central Valley and especially in Kern County. It was there where, many decades earlier, the disease known as San Joaquin Valley fever was first discovered to be due to a fungus [38,39]. In the 1990s, a consortium of five laboratories agreed to work together on the problem. This willingness to collaborate combined with the importance of CM as a California public health problem persuaded the California Health Care Foundation and the California state legislature to provide funds. As detailed elsewhere [40], the collaboration, known as the Valley Fever Vaccine Project, was very productive, and two vaccine candidates resulted from the work. One was a chimeric recombinant peptide formulated with monophosphoryl lipid A, a TLR4 adjuvant that stimulates a Th-1 biased response [41]. Although promising, it encountered manufacturing obstacles, and the adjuvant became commercially unavailable at the time [42]. The other vaccine candidate was a genetically modified CM strain [43] with reduced virulence. Its potential as a clinical vaccine was not pursued. However, even more significant than the Valley Fever Vaccine Project’s own specific accomplishments was that its investigators accessed the explosion of new biologic information and experimental technologies. These resources, now within reach of the coccidioidal vaccine effort, enabled the subsequent discovery of the vaccine candidate discussed for the rest of this review.

## 4. The Discovery of *CPS1* as a Critical Gene for Spherule Maturation

**Figure 2:**
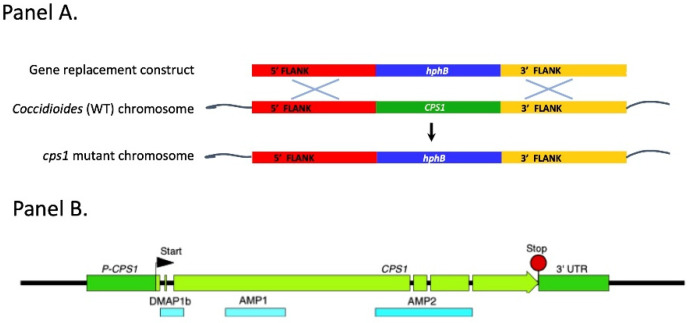
**Panel A:** The *CPS1* gene replacement construct was introduced into *Coccidioides posadasii* strain Silveira via Agrobacterium-mediated transformation homologous recombination between the construct and the chromosome results in a *C. posadasii* strain that completely lacks the coding sequences of the *CPS1* gene. **Panel B:** Suggested functional domains of Cps1.

Research into the virulence of a maize fungal pathogen, *Cochliobolus heterostrophus*, led to the identification of *CPS1* as contributing to its pathogenicity [44]. *CPS1* is a member of the *DIP2* (Disco interacting protein 2) gene family found from fungi to humans [45,46,47]. *DIP2* genes share an N-terminal protein interactions domain, DMAP1b, and two catalytic adenylation domains of the AFD class I superfamily. In Drosophila and mice, DIP2 members are functionally critical for proper brain neuron development and are proposed to work via binding AMP and catalyzing ATP-dependent acyl-CoA formation. The role of Cps1 in the *Coccidioides* life cycle is not yet understood. However, because *C. hetereostrophus CPS1* is a pathogenicity factor, as well as data indicating that *C. posadasii CPS1* expression was up regulated in early spherulation led to testing whether *C. posadasii CPS1* may also be important for virulence. To address this question, a complete gene replacement mutant (*Δcps1*) was created in strain Silveira [48] (Figure 2A).

*Δcps1* displayed only modest changes in hyphal growth and sporulation. Maturation of arthroconidia on solid media was delayed and the yield of spores was reduced compared to the wild-type parent [47]. *C. posadasii* strain Silveira (WT) produced 2.3 × 10^8^ cfu per agar plate after four weeks compared to 1.4 × 10^8^ cfu of *Δcps1* after six weeks, but arthroconidia of the mutant and parent were otherwise similar.

**Figure 3:**
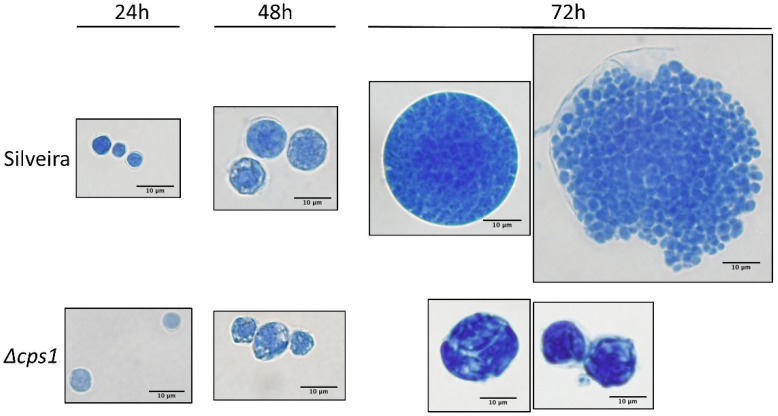
In vitro spherules of the *C. posadasii* strain Silveira and the Silveira *Δcps1* mutant. Silveira and *Δcps1* arthroconidia were grown shaking in RPMI media at 37 °C with 20% CO_2_, and assessed every 24 h. Silveira spherules enlarge, develop a thick wall and by 72 h begin to lyse and release endospores that begin a second round of spherulation (not shown). *Δcps1* spherules also begin to enlarge, but have thin walls, and by 72 h begin to undergo plasmolysis, failing to mature and produce endospores. Spherules are stained with lactophenol blue.

In contrast to the hyphal growth phase, there were striking differences in the in vitro growth of spherules. Shown in Figure 3, WT arthroconidia differentiate into spherules that endosporulate and are released within 96 h. In contrast, *Δcps1* arthroconidia round up and initiate spherulation by 24 h, but by 48 h are irregularly shaped with thin walls, and undergo plasmolysis by 60–72 h. We believe that this initiation of spherulation may be critical to providing a protective vaccine because another spherulation-defective mutant we created, *Δryp1*, produces arthroconidia that fail to initiate spherulation and does not provide protection against subsequent WT infection [49]. As predicted by the in vitro studies, spherules nearly completely fail to persist in vivo (see below).

We are currently taking molecular genetic, structural biologic, and biochemical approaches to determine why Cps1 is so critical for spherule development. We have shown that the phenotypic defects of *Δcps1* are due to the deletion since complementation of *Δcps1* with the WT gene restored full virulence and WT in vitro spherulation (Mandel, unpublished). Searching the NCBI conserved domains database with Cps1 (https://www.ncbi.nlm.nih.gov/Structure/cdd/wrpsb.cgi (accessed on 17 March 2021)) identified three domains, the N-terminal DMAP1 binding domain, and the two adenylate forming domains of the Class I superfamily (Figure 2B). Preliminary data suggest that the latter two domains are critical for spherulation in *Coccidioides,* while the DMAP1b domain is not. Although initial in silico analyses indicated that Cps1 was a transmembrane protein, which was consistent with reports for *Drosophila* and human members of the DIP2 family of proteins [45,46,47], more recent in silico analysis of Cps1 using AlphaFold 2.1 [50] on our local workstation (https://www.deepmind.com/research/highlighted-research/alphafold (accessed on 17 March 2021)) predicts Cps1 to be a peripheral membrane protein. Overexpression of Cps1 in yeast and purification of the protein from a membrane preparation by high salt extraction supports this prediction (Seka and Tomasiak, unpublished). Purification of the protein will allow a detailed structural definition of key domains and the testing of whether the adenylate forming domains are able to catalyze ATP-dependent production of acyl-CoA bioproducts, as suggested for Drosophila Dip2, where Dip2 deletion resulted in significant reduction of acyl-CoA levels [44]. Cps1 could potentially play a role in proper membrane structure during spherulation, and deletion would result in membrane defects that could lead to the collapse of developing spherules. If so, further studies are needed to define why the defect results in only a dramatic effect during the parasitic but not the saprobic phase. With construction of domain deletion strains and the purification of the Cps1 protein, these questions are within reach.

## 5. The Pre-Clinical Safety Profile of *Δcps1*

**Figure 4:**
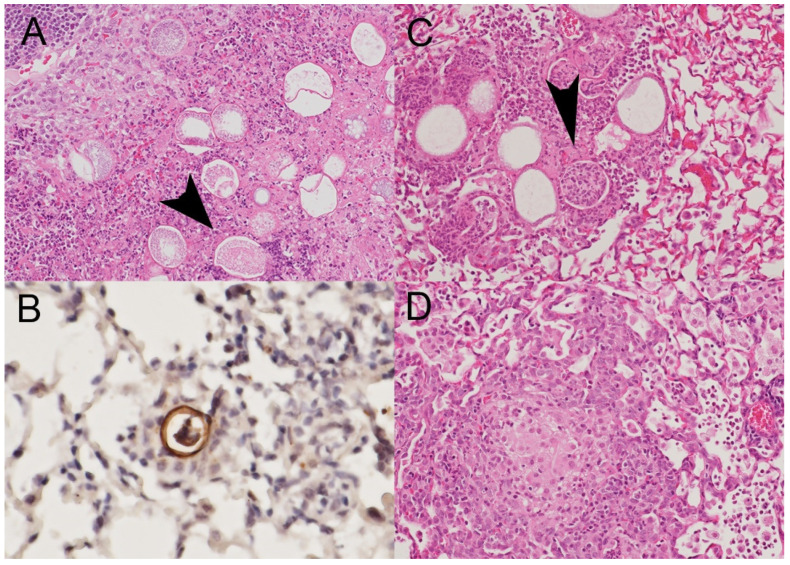
(**A**) Day 15 post-infection, *C. posadasii* strain Silveira (WT) has many large spherules densely filled with endospores (arrowhead). (**B**) On day 3, *Δcps1* spherules have few or no endospores, their walls are irregular, and there are neutrophils rather than endospores in the degrading organism. (**C**) Day 4, *Δcps1* spherules show numerous spherules devoid of endospores and often filled with neutrophils (arrowhead). (**D**) Day 10 of infection, small residual granulomas which have no spherules observed. (**A**,**C**,**D**)—hematoxylin and eosin stain, 200 × magnification; (**B**)—*Coccidioides*-specific stain for Ag2/PRA, 400 × magnification).

C57BL/6 (B6) mice, both B6/J and B6/N, are very sensitive to many strains of *Coccidioides* spp [51,52], and in our hands the LD_95_ for intranasal (IN) infection of strain Silveira is less than 50 arthroconidia, with most mice succumbing within two to three weeks. However, B6 mice infected with up to 4400 spores of *Δcps1* IN survived the duration of the experiment (two weeks), and cultures of virtually all lungs from these mice yielded no residual *Δcps1* organisms. BALB/c, which are at least as susceptible to IN coccidioidal infection [53], were administered 10,000 to 25 million spores and pairs of animals sacrificed for histopathology at different intervals up to 10 days following infection. The very highest inoculum produced transient suppurative bronchial and alveolar infiltrates seen best histologically on day 3. However, endospores were seen only infrequently, suggesting little if any propagation. By days seven and 10, only small sporadic well-organized granulomas were observed, the number of contained spherules were less than 10% of the numbers seen earlier, and sometimes the spherules were empty. Representative differences in tissue spherules between Silveira (WT) and *Δcps1* are shown in Figure 4.

Additional and dramatic evidence for safety was obtained by IN infection of NOD.Cg-*Prkdc^scid^IL2rg^tm1Wjl^*/SzJ (NSG) mice, which lack mature T cells and B cells. Also, because they lack the IL-2 receptor common gamma chain, they are deficient in NK cells [54]. When four of these mice were infected with 1030 *Δcps1* arthroconidia IN, all survived until the planned termination of the experiment at two weeks. Two of these mice and two others sacrificed at six days revealed no spherules in lung sections. Of two more mice sacrificed for fungal culture at two weeks, one was negative and the other showed 8200 cfu, indicating that persistence of some viable *Δcps1* is possible without evident disease in severely immunodeficient animals.

One of the most appropriate and reliable models for human vaccines is the pig whose physiology and immune system have considerable similarity to humans [55,56,57,58,59,60,61]. For this reason, we evaluated the tolerance of *Δcps1* in four to six-week-old Yorkshire piglets (three pigs per treatment), evaluating three subcutaneous (SC) administrations for injection site tolerance and safety. Arthroconidia of *Δcps1* were administered on days 0, 14 and 28 at one of four doses (10,000, 100,000, 250,000 and 500,000 arthroconidia). Pigs given 250,000 viable spores that were at potencies of 10%, 50% or >90% viability, while the other three groups were held constant at >90% viability. Injections were well tolerated with no discomfort noted and with no apparent adverse events from the increasing dose of nonviable arthroconidia. All piglets gained weight during the study. Swelling at injection sites was recorded in less than 25% of the animals, averaging 2–3 cm^3^, more frequent with the higher inoculum, and more commonly in primary than in booster vaccination sites. All resolved by day 42 when the study was ended. Injection sites were microscopically normal with no inflammatory cells in 29 of 45 examined. The 16 others showed small areas of lymphocytic or neutrophilic inflammation, no fibrosis, and no spherules or endospores. For the 90% viability group, cultures from injection sites at day 42 were positive from four of 17 primary sites, two of 14 of initial boost sites, and 0 of 16 second boost sites. At the lower viabilities, the secondary boost sites were also negative and fewer primary boost sites were positive. Overall, the tested vaccine candidate injection site reactivity was low and well tolerated, and no systemic clinical signs were observed.

## 6. Vaccination with *Δcps1* Provides Robust Protection against Experimental Pulmonary Coccidioidal Infection in Mice and Dogs

**Figure 5:**
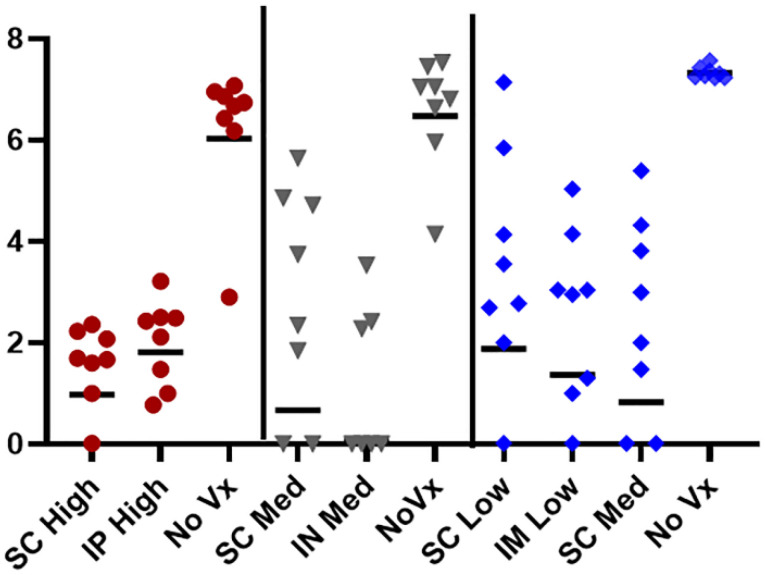
Protection afforded to B6 mice by *Δcps1* vaccination by different routes, intranasally (IN), intraperiotoneally (IP), subcutaneously (SC), or intramuscularly (IM). Vaccine with viable *Δcps1* arthroconidia: High = 25,000–50,000; Med = ~10,000; Low = 1000–2000; No Vx = saline control. Mice were vaccinated twice two weeks apart and challenged with approximately 100 arthroconidia of Silveira (WT). Vaccinated groups had significantly reduced lung fungal burden (*p* < 0.05 compared to controls) two weeks post-infection. Colors/symbols denote different studies.

Numerous vaccine trials with *Δcps1* have been conducted in mice. The original publication [48] described that *Δcps1* vaccination (prime and boost) of B6 mice with 50,000 spores in each injection, either SC or intraperitoneally (IP), resulted in uniform survival, and lung fungal burden was three logs less than that of mice vaccinated with the recombinant chimeric vaccine previously developed by the Valley Fever Vaccine Project [41], and five logs less than sham vaccinated mice. In additional studies, this was extended to IM vaccinations (Figure 5). Vaccination of BALB/c mice with *Δcps1* SC resulted in 19 of 20 mice surviving until the end of the experiment, day 28 after infection, with 46 *C. posadasii* Silveira spores. In contrast, control mice that received the chimeric antigen vaccine or adjuvant alone all died by day 15 post infection IP. The lung fungal burdens of surviving *Δcps1*-vaccinated mice was less than 1000 cfu per lung for 18 of 19 mice, with 7 producing no growth from their lungs. Moreover, the spleen of only one mouse of the *Δcps1*-vaccinated mice grew colonies of Silveira (WT), whereas spleens of all control mice were uniformly infected. In a second report [62], these observations were extended to demonstrate that protection from *Δcps1* vaccination was unchanged if the challenge infection was delayed up to six months if vaccinated mice were observed for up to six months after challenge, or if WT coccidioidal infection was done with a strain of *C. immitis*. Viable spores are required for protection, since spores killed by either radiation or ethanol treatment were ineffective. Not all avirulent mutants afford protection. For example, vaccination of mice with a *RYP1* (Required for yeast phase) knockout (*Δryp1*), which is also avirulent in mice, yielded no protection from WT challenge [48]. Since *Δryp1* does not initiate spherule development, it is possible that this step, which *Δcps1* does, is needed to stimulate protection.

These encouraging findings led the investigative team, in partnership with Anivive Lifesciences, to initiate a program to develop a clinically useful vaccine for dogs and a vaccine/challenge study was performed [63]. Thirty young adult male and female beagle/beagle mix dogs were vaccinated SC twice 4 weeks apart with 10,000, 50,000 or 100,000 arthroconidia, or 100,000 arthroconidia only once, or saline as a control (n = 6/group). Approximately four weeks later, dogs were challenged with 10,000 virulent arthroconidia of *C. posadasii* by aerosol nebulization of a saline suspension. Dogs were monitored for 8 weeks, with daily clinical monitoring of health, and biweekly assessment of CBC, serum chemistries, lung radiographs, and serum antibodies. At the end of the study, lungs were evaluated grossly, by fungal culture, and by histopathology of 1 cm^3^ specimens from each lobe and a mediastinal lymph node. At all three doses, dogs vaccinated twice had minimal lung and lymph node fungal burdens, few radiographic or histopathological abnormalities, and low or no antibody titers, with similar low composite disease scores for the all vaccinated groups, means ranging from 9.5 to 11.7, compared to 123.7 for unvaccinated controls) [62]. Dogs given a single high dose of the vaccine had higher fungal burdens and composite disease scores (mean, 55.9) than prime/boost dogs and they were not significantly different from controls [63]. One dog in the single dose vaccination group showed a lesion in a rib bone, which served as further evidence that protection from a single vaccine dose was incomplete.

This study demonstrates the efficacy of the *Δcps1* vaccine to mitigate infection in a target species, which is also a larger animal model that has a rate and range of naturally acquired disease similar to humans [64]. The dogs had transient injection site reactions to the SC administration of the vaccines, which were clinically resolved by six weeks after primary inoculation and could not be identified histologically at necropsy approximately four months after vaccine administration. Thus, *Δcps1* appears to be both safe and efficacious in dogs, a huge step toward developing this vaccine for use in humans.

## 7. Mechanisms of Protection

The underlying premise that drives the effort to develop a preventative vaccine for CM is the lifelong protection that natural coccidioidal infection affords to individuals from illness if an arthroconidium is inhaled again. Although incompletely understood, the protective immunity from naturally acquired infection is generally understood to be a cellular response [65,66]. Studies thus far suggest that this is the case also for the enduring protection that results from vaccination with *Δcps1*. Cytokines from infected lungs of vaccinated mice in the first four days after WT *C. posadasii* challenge showed significant increases of IFN-γ production; additionally, an IFN-γ recall response to spherule lysate could be elicited from CD4+ spleen cells [62]. Of particular interest, mice with deleterious mutations in a number of immunologically important genes were found to be at least partially protected by *Δcps1* vaccination, although Rag deficient mice were not [67,68]. This suggests that in addition to CD4-mediated protection there are likely other pathways such as one mediated by CD8 [25] that can mediate protection, an observation that raises the hope that the *Δcps1* vaccine might protect patients who are susceptible to disseminated CM because of subtle and complex immunogenetic dysregulation of innate responses to coccidioidal infection [69].

**Figure 6:**
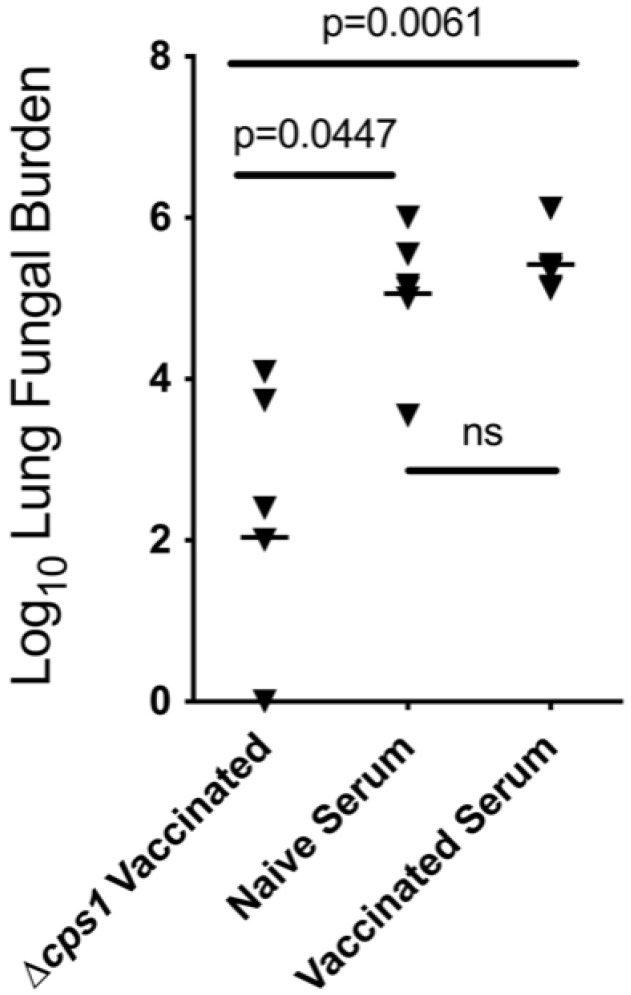
Serum from naïve or *Δcps1*-vaccinated mice was transferred (500 µL/mouse IV) into naïve mice 24 h before IN challenge with C. posadasii strain Silveira. Two weeks later, lung fungal burdens were quantitated from serial dilution of lung homogenates. There is no protection afforded by the transfer of immune serum. *Δcps1*-vaccinated mice were infected as controls for the reduction of fungal burden.

**Figure 7:**
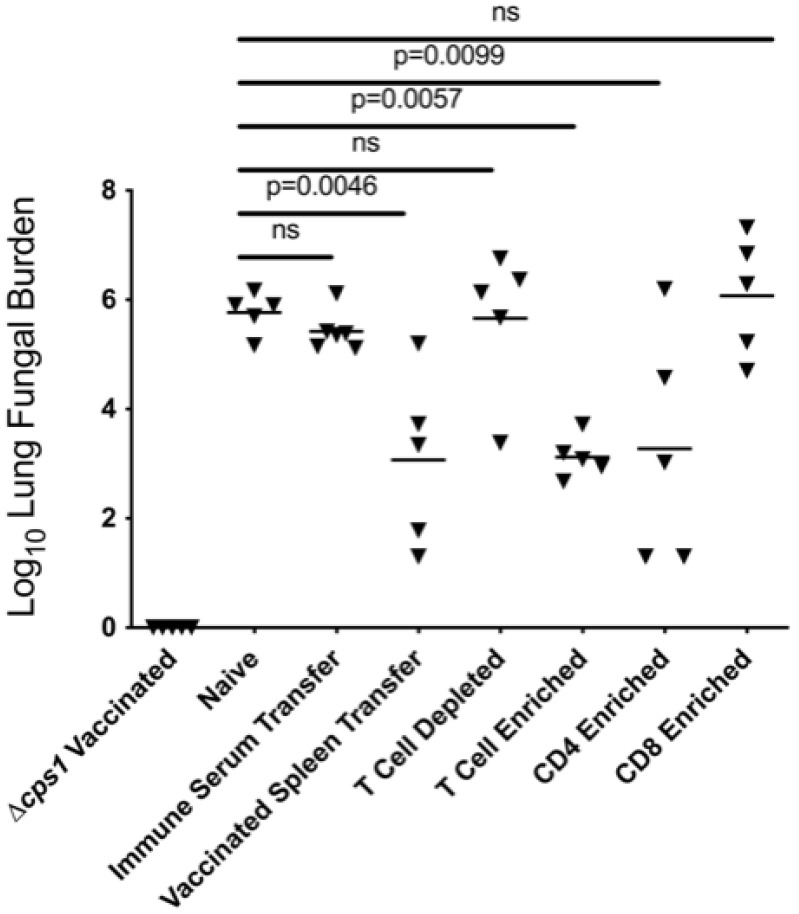
Total splenocytes from naïve or subsets from *Δcps1*-vaccinated mice were transferred IV (1:1 donor: recipient) into naïve mice 24 h before IN challenge with *C. posadasii* strain Silveira (WT). Two weeks later, lung fungal burdens were quantitated from serial dilution of lung homogenates. Protection compared to naïve animals was analyzed by one-way ANOVA with Dunnett’s correction for multiple comparisons. *Δcps1*-vaccinated mice were infected as controls for reduction of fungal burden.

To further elucidate the mechanisms of protection imparted by the *Δcps1* vaccine, a variety of adoptive transfer experiments have been carried out in mice. Sera from vaccinated animals have high levels of IgG that recognized both spherule lysate as well as the protein Cts1, the active protein in the clinical complement fixation test [69]. To determine if these sera could provide passive protection, serum from *Δcps1*-vaccinated mice was transferred to naïve mice via tail vein injection (~500 µL/mouse). The following day the mice were challenged IN with strain Silveira. Fourteen days after challenge, mice were sacrificed and lung fungal burden was determined by serial dilutions and culture. These mice had burdens approaching 10^6^ cfu/lung, similar to mice receiving naïve serum (Figure 6), and very different from mice actively immunized with *Δcps1,* which had a low fungal burden in the lungs. This result indicates that antibodies alone are not protective. To determine if immune cells from vaccinated mice could provide passive protection, splenocytes (1:1 ratio donor to naïve mice) were transferred one day before challenge. When the recipient mice were sacrificed 14 days later, they had a reduced lung fungal burden as compared to naïve mice (Figure 7). Though not as low as the actively *Δcps1*-vaccinated animals, it still indicates that cellular immunity is involved. In an additional experiment, we used magnetic beads to deplete T cells from the splenocyte preparation before transfer. When this T-depleted splenocyte mixture was transferred, the fungal burdens were indistinguishable from the unvaccinated mice, highlighting the role of T cells in the protection imparted by *Δcps1* vaccination. This was further strengthened by transferring just the CD3+ fraction of splenocytes (T cell enriched). Transfer of just the T cell enriched fraction yielded fungal burdens similar to transferring whole vaccinated splenocytes. Furthermore, the transfer of CD4-enriched splenocyte fractions provided similar protection to whole immune splenocytes, highlighting that the protection from *Δcps1* vaccination can involve CD4+ T cells (Figure 7).

The exact mechanism through which the CD4+ T cells are providing protection is unclear. They may enhance the recruitment of other immune effectors to the site of infection or help refine the existing immune response by amplifying signals or even controlling inflammation through Treg activity. Whether these CD4+ T cells need to make a specific cytokine or combination of cytokines has not been explored, and in many vaccines poly functional CD4+ T cells are much more effective at providing protection compared to cells making a single cytokine. Perhaps we could see increased protection in the passive transfer if we used lung T cells for the transfers; these resident cells may home back to the lung more effectively or have a different phenotype than those from the spleen. These questions open an interesting avenue for further exploration.

Initial studies of *Δcps1* vaccine demonstrated similar levels of protective efficacy when spores were inoculated SC or IN [48]. There may even be a trend toward greater efficacy by the IN route. This raises questions about how the vaccine mode of action might differ according to the site of inoculation, especially when given at the site of primary infection e.g., the lung. For example, what is the role of lung-resident T cell memory (T_RM_) in vaccine resistance, as compared to migratory T cells? In work on the related endemic dimorphic fungus, *Blastomyces dermatitidis,* migratory CD4+ T cells from the spleen and draining lymph nodes are responsible for vaccine resistance against lethal pulmonary challenge in mice when a subunit vaccine is given SC [70]. Conversely, it is likely that T_RM_ rather than migratory T cells confer resistance in mice vaccinated intranasally against CM, although this has not been studied. Since the lung epithelium plays an important role in restraining the related inhaled dimorphic fungi *Histoplasma capsulatum* and *B. dermatitidis*, such cells (or other lung stromal elements) may also have a role in promoting vaccine resistance when *Δcps1* is delivered IN, either in eliciting T_RM_ or in maintaining them long term.

The relative roles of T_RM_ vs. migratory T cells in vaccine resistance could be tested by parabiosis where the circulation (and migratory cells) is shared between conjoined mice, but the lung (and T_RM_) is not shared. Thus, if one of the mice in a pair is vaccinated, migratory cells would circulate between them and protect the other mouse, whereas T_RM_ could not circulate and protect the unvaccinated, experimentally challenged conjoined mouse. An alternative approach to answering this question of T_RM_ vs. migratory cells involves the adoptive transfer of cells from either compartment of vaccinated mice into unvaccinated recipients.

The latter strategy (adoptive transfer) was used effectively in mice given a subunit vaccine against blastomycosis—*Blastomyces* endoglucanase 2 (Bl-Eng2). Transfer of antigen specific T cells when the antigen is known can enhance the resistance phenotype. For example, the T cell epitope in Bl-Eng2 was mapped and peptide-MHC II tetramers were created so that antigen specific T cells could be tracked, analyzed, harvested and transferred. Identification of the immunodominant antigen(s) in *Δcps1* antigen(s) that mediate the vaccine effect could likewise enable similar studies and analyses. Should such an immunodominant antigen itself be immunogenic and protective, that antigen would offer value for analyzing and characterizing correlates of vaccine immunity in both mice and humans and could be useful as a component in a subsequent subunit vaccine.

In contrast to the murine model of CM, mice vaccinated IN against blastomycosis with either an attenuated strain or a subunit antigen fail to acquire resistance [71,72]. Failure of IN *Blastomyces* vaccine stems from poor priming of antigen-specific CD4+ T cells (live-attenuated vaccine) or deviation of CD4+ T cell phenotype toward Treg cells or sharply polarized Th17 cells (Bl-Eng2 subunit vaccine) rather than a mix of Th1 and Th17 cells in the protective phenotype following SC vaccination. The use of Bl-Eng2 specific tetramers enabled analysis of the phenotypic properties of antigen specific T cells elicited by the SC route compared to the IN route. Tetramer-positive T cells analyzed after vaccination by each route have also revealed additional transcriptional features of protective CD4+ T cells and potentially novel populations of cells linked with protection.

## 8. Development Plan to Initiate Human Studies

Because of the limited and regional market size for a preventive vaccine against CM, there is little or no interest among large biopharmaceutical companies. However, smaller specialty pharma and biotechnology companies have expressed interest in such a product, particularly since a well-defined vaccine candidate is available and proof of concept was achieved for the veterinary indication. In addition, the public health impact of the disease warrants support from federal and state institutes of government. The possible addition of CM to the list of products eligible for a Priority Review Voucher from FDA would be a major motivating factor to get private industry to invest. At this point in time, at least one biotechnology company (Crozet BioPharma) has expressed a strong interest in undertaking the development of *Δcps1*.

Approximately half of currently approved human vaccines are live-attenuated vaccines, attesting to the robust and durable humoral and cellular immune responses elicited by these vaccines. However, there is no precedent for a live-attenuated eukaryotic human vaccine, let alone for a fungal disease specifically. For these reasons, the principal challenge for translation of the encouraging data described in rodent, pig and canine models to humans will be the demonstration of safety. Other challenges include the development of a consistent biomarker of protective immunity in humans, presumably by measurement of T cell responses, and the execution of randomized, controlled trials (RCTs) demonstrating vaccine efficacy (VE) for a prespecified primary endpoint and prespecified lower bound of the 95% confidence interval. The latter benefit, weighed against any safety risks defined in a trial showing a low risk (e.g., <10^−4^) of significant adverse events, will be critical in obtaining a recommendation for use from the Advisory Committee on Immunization Practices (ACIP).

A final challenge is the manufacture and control of a purified arthroconidial formulation according to current good manufacturing practices (cGMP). Vaccine manufacturers who have multi-use facilities are reluctant to deal with the difficulty and risk of environmental separation and control of spore-forming organisms such as *Δcps1*. Fortunately, there is a robust capability for process development and cGMP manufacturing of spore-forming bacterial vaccines, use of spore-formers for production of recombinant products, and live biotherapeutics. An experienced contract development and manufacturing partner has already been identified.

**Figure 8:**
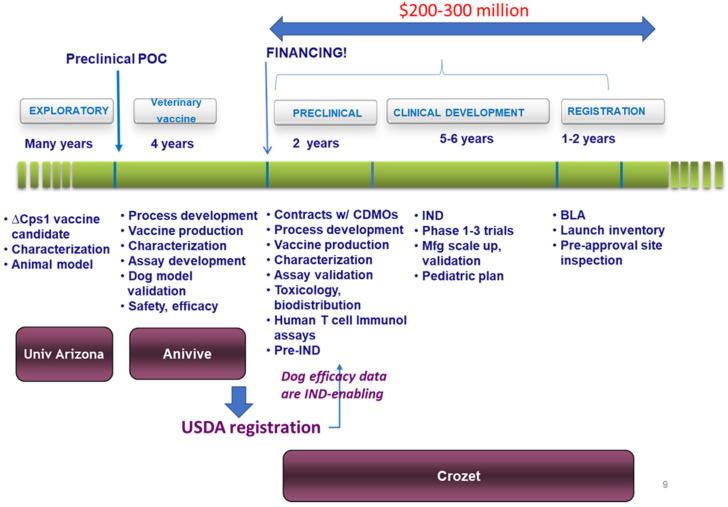
Schematic of the development pathway and timelines for *Δcps1* vaccine.

However, all of these challenges are considered to be surmountable, and a development plan, with schedules and budgets, has been produced, with the expectation that a *Δcps1* vaccine could reach marketing authorization within 8–10 years (a typical timeline for development of vaccines in other than extraordinary emergencies, such as SARS-CoV-2 and Ebola) and at reasonable cost (Figure 8). The relatively low cost reflects the fact that years and substantial resources have already been invested in development of the *Δcps1* vaccine candidate and in bringing the canine vaccine close to regulatory approval by the U.S.D.A.

Whereas *Δcps1* may be the first fungal vaccine for humans, it would be only one of many live-attenuated vaccines. In fact, of 28 vaccines registered for human use in the US or other countries, 15 (54%) are live-attenuated (viral or bacterial) vaccines. Of these, virtually all are replication competent and depend on the multiplication of their antigenic mass in vivo. As pointed out earlier, *Δcps1* may have very limited capacity to replicate in vivo, and behaves like a live, defective vaccine such as the modified vaccinia Ankara or non-replicating adenovirus vaccines. This is an important safety consideration. Another advantage of live vaccines, including *Δcps1*, is that they do not need an additional adjuvant for their activity and generally provoke a Th-1 cytokine orientation, robust cellular responses, and durable memory without the need for frequent boosting.

Development of the vaccine candidate for humans will require a series of well-designed nonclinical studies conducted in accordance with current Good Laboratory Practices (cGLP) to extend existing published data and demonstrate lack of toxicities including detailed histopathological evaluation, in a relevant animal species, as well as a study on biodistribution and persistence. It is very possible that such studies will need to be performed in nonhuman primates. In addition to safety, the immunological assays to support human studies can be developed, since T cell immunologic methods are well established for nonhuman primates. Evidence from these studies, combined with existing safety and efficacy data in dogs, will enable entry to Phase 1 and 2 clinical trials, which will provide critical information on safety, dose, schedule, and immunogenicity of the *Δcps1* vaccine in preparation for pivotal trials. The traditional regulatory pathway for *Δcps1* will be required, as the incidence of the disease is sufficiently high and clinical infection and disease endpoint(s) are sufficiently defined to enable a Phase 3 RCT.

## 9. Conclusions

As reviewed here, there is a compelling rationale to discover a preventative vaccine to manage the growing problem of CM. This has been pursued for nearly seven decades. An early killed spherule vaccine was ineffective and too irritating. A promising recombinant vaccine was unable to be produced because of technical limitations, and further research into synthetic vaccines on a variety of platforms hold promise for the future. In the meantime, the live-attenuated *Δcps1* vaccine has emerged as a potentially safe and effective candidate. The *Δcps1* vaccine is being actively developed for veterinary use, and as detailed here, it has a strong foundation to support its continued development for humans. The public health and economic impact of CM amply justify such a campaign. However, finding the needed resources to develop a vaccine for an orphan disease will be a challenge without significant support from the public sector, either from federal agencies or the states where the problem is most endemic. In the pursuit of this vaccine for clinical use, it is entirely possible that limitations may be discovered that preclude its further development. Even if that is the case, using the *Δcps1* vaccine to again open an FDA IND will set in motion the process of vaccine development for CM which has been dormant for many years. This would then also support any future vaccine that might turn out to be more attractive. Moving forward with the *Δcps1* vaccine and continued research into other vaccine strategies would both be very useful strategies to manage CM.

## Data Availability

Data for studies shown are available from the authors upon request.
